# Association between -T786C NOS3 polymorphism and resistant hypertension: a prospective cohort study

**DOI:** 10.1186/1471-2261-9-35

**Published:** 2009-08-04

**Authors:** Ignacio Cruz-González, Esther Corral, María Sánchez-Ledesma, Angel Sánchez-Rodríguez, Cándido Martín-Luengo, Rogelio González-Sarmiento

**Affiliations:** 1 Cardiology Department, University Hospital of Salamanca, Salamanca, Spain; 2 Molecular Medicine Department, University of Salamanca, Salamanca, Spain; 3 Internal Medicine Department, University Hospital of Salamanca, Salamanca, Spain; 4 Instituto de Biologia Molecular y Celular del Cancer (IBMCC), BMCC, University of Salamanca- CSIC, Salamanca, Spain

## Abstract

**Background:**

It is estimated that 5% of the hypertensive patients are resistant to conventional antihypertensive therapy. Polymorphisms in the endothelial nitric oxide synthase (NOS3) gene have been associated with high blood pressure levels, but not with resistant hypertension. The aim of the present study was to investigate if the -786T>C and G894T (Glu298Asp) polymorphisms of the NOS3 gene were associated with resistant hypertension.

**Methods:**

A prospective case-control observational study was performed. From a series of 950 consecutive patients followed up during 42 months, 48 patients with resistant hypertension were detected. 232 patients with controlled high blood pressure were also included.

**Results:**

No differences were observed in the distribution of G894T (Glu298Asp) NOS3 genotypes between the resistant hypertension group and the controlled hypertension patients. However, genotype -786CC was more frequent in the group of patients with resistant hypertension (33.3%) than in the group of patients with controlled high blood pressure (17.7%) (p 0.03). Furthermore carriers of allele T (-786TC and -786TT) were more frequent in patients with controlled hypertension (82.3%) than those with resistant hypertension (66.7%) (Multivariate analysis; RR 2.09; 95% CI 1.03–4.24; p 0.004).

**Conclusion:**

Our results indicate that genotype -786CC of the NOS3 gene increase the susceptibility to suffer resistant hypertension, which suggest that resistance to conventional therapy could be determined at the endothelial level.

## Background

Hypertension may be termed resistant when a therapeutic plan that has included attention to lifestyle measures and the prescription of at least three drugs including a diuretic in adequate doses has failed to achieve goal blood pressure (BP) defined as a systolic/diastolic pressure of < 140/90 mmHg or < 130/80 mmHg in diabetics patients [1]. The prevalence of "true" resistant hypertension (excluding secondary causes of hypertension, white coat hypertension, inadequate doses, etc) is less than 5% of the general hypertension population [2]. In general, resistant hypertension has been reduced to an issue of treatment adherence or suboptimal treatment.

Genetic determinants of human hypertension are poorly understood although polymorphisms in several genes have been associated with elevated blood pressure levels. However, there are only few studies that consider resistant hypertension from a genetic point of view [3-6].

Causative genes for essential hypertension have been identified through candidate gene approach. Among the genes investigated, the endothelial nitric oxide synthase gene (NOS3) has drawn considerable attention because of its substantial contributions to BP regulation. Endothelial nitric oxide synthase (eNOS) mediates the release of nitric oxide, a potent vasodilator, from endothelial cells; and the disruption of NOS3 gene leads to hypertension in mice [7]. Several lines of evidence have also supported that impaired nitric oxide production directly leads to an elevation in BP [8]. Hence, several investigators have examined the NOS3 gene as a potential candidate for essential hypertension, but its relation with resistant hypertension has not been verified.

The aim of the present study was to investigate if the promoter (-786T>C) and exon 7 (G894T/Glu298Asp) polymorphisms of the eNOS gene are associated with resistant hypertension and essential controlled hypertension.

## Methods

### Study subjects

An observational prospective case control single centre study was performed. Once excluded the secondary causes of hypertension and those patients who did not adhere to lifestyle measures, patients were considered to have resistant hypertension when a therapeutic plan that included lifestyle modification (including smoking cessation and achieving a body mass index at least < 30 kg/m2) and prescription of at least three drugs including an adequately dosed diuretic, failed to achieve the target BP of < 140/90 mmHg (< 130/80 in diabetics) on any occasion during follow-up. These patients were controlled at least every month, 24 h-Ambulatory Blood Pressure were determined twice and they never achieve goal BP. Treatment adherence was confirmed in all the patients with analytic control and adherence tests. Secondary causes of hypertension as obstructive sleep apnea, primary aldosteronism, thyroid diseases, chronic kidney disease, pheocromocytoma, Cushing's syndrome and aortic coarctation were excluded by clinical and laboratory evaluation (urinalysis, complete blood cell count, blood chemistry profile) [1]. Further evaluations were performed if a secondary cause of hypertension was suspected. Pharmacological treatment of each patient was reviewed looking for pharmacological agents that could produce transient or persistent increases in BP (Nonsteroidal anti-inflammatory drugs, sympathomimetics, cocaine, amphetamines, other illicit drugs, oral contraceptive hormones, adrenal steroid hormones, erythropoietin, cyclosporine, tacrolimus, licorice, over-the-counter dietary and herbal supplements).

Controlled hypertension population was defined as having a systolic/diastolic BP of > 140/90 mmHg (> 130/80 mmHg if diabetics) on three separate occasions but controlled (< 140/90 mmHg or < 130/80 mmHg if diabetics) after treatment during the follow-up.

Of 950 Caucasic hypertensive subjects consecutively referred to the Hypertension Unit of the University Hospital of Salamanca during 42 months, 48 resistant hypertensive patients (5.2%) were identified. After statistical estimation, a total of 232 Caucasics age and sex matched hypertensive subjects who had achieve BP goals after treatment were also included in the study. 110 healthy subjects were examined to determine the allelic distribution in a Caucasian control population.

Patients were seated quietly for at least 5 minutes and blood pressure measurements were performed at least twice using a Hawksley random zero mercury sphygmomanometer with an appropriate cuff size, and verified three times with an Omron Automatic Blood Pressure Monitor, Model HEM-773AC (Omron HealthCare Inc, Bannockburn, Illinois USA), the mean value of the manual measures were used for the analysis. Ambulatory blood pressure monitoring (ABPM) (24-hour) was measured using an Oscar ABPM system (SunTech Medical Inc., Morrisville, NC, USA). Target organ damage was assessed by both echocardiograph and fundoscopy by a single-operator blinded to the patient's condition. Degree of left ventricular hypertrophy (LVH) and retinopathy were determined. Significant retinopathy was defined as grade ≥ 2 and LVH as left ventricular mass > 125 g/m^2 ^in men or 110 g/m^2 ^in women. All patients were treated according to the recommendations of the Joint National Committee 7 [1]. Informed consent was obtained from all subjects. The study was approved by the Ethical Committee of University of Salamanca and was in accordance with the principles of the Declaration of Helsinki.

### Genotyping for eNOS Gene Polymorphisms

Genomic DNA was isolated from peripheral blood leukocytes as previously reported [9]. The presence of the -786T>C and G894T alleles was determined by polymerase chain reaction -restriction fragment length polymorphism (PCR-RFLP) analysis. PCR was performed with primer pairs designed to amplify either a 180-bp segment containing the promoter (-786T>C) variant (sense: 5' TGGAGAGTGCTGGTGTACCCCA 3'; antisense: 5' GCCTCCACCCCACCCTGTC 3') or a 200-bp segment encompassing exon 7 (sense: 5' AACCCCCTCTGGCCCACTCCC3'; antisense: 5' TCCATCCCACCCAGTCAAT 3'). The PCR fragment containing the -786T>C polymorphism was subjected to digestion with MspI, which cuts the PCR product when the T at position -786 is replaced by a C. The fragment containing exon 7, was digested with MboI, which cuts only in the presence of T at position 894 (corresponding to Asp298). Digested samples were separated on a 3% ethidium bromide-stained agarose gels and visualized by UV transillumination.

### Statistical analysis

All genotype groups obeyed the Hardy-Weinberg equilibrium. Normally distributed parametric data were expressed as mean ± SD, and compared with Student's t-test. The results obtained for the different groups were compared using chi square and Fisher's exact test. Logistic regression, including age and sex as confounding factors, was applied to evaluate differences in allele frequencies between subjects. A p-value < 0.05 was regarded as significant. Odds ratio and 95% confidence intervals (OR; 95% CI) were also calculated. Sample size and statistical power were estimated according to the preliminary results obtained in this study. Sample size was estimated for a 95% confidence intervals and 80% statistical power. All analyses were performed using Statistical Package for Social Science (SPSS Inc, Chicago, Illinois, USA) version 13.0.

## Results

The distribution by age and sex was similar in the group of patients with resistant hypertension and in the group of patients with controlled hypertension. The characteristics of the population are shown in Table 1. Retinopathy (grade 2 or more) and left ventricular hypertrophy were more frequent in resistant hypertension patients (80% vs 53% p 0.02; 75% vs 44% p < 0.001). A longer hypertension evolution was observed in the group of patients with resistant hypertension (17.05 ± 12 vs 8.61 ± 8 years; p < 0.001).

**Table 1:** Demographic characteristics in hypertensive patients (resistant and controlled) and in healthy controls

	***RESISTANT HYPERTENSION*** ***(n = 49)***	***CONTROLLED HYPERTENSION*** ***(n = 232)***	***HEALTHY CONTROLS*** ***(n = 110)***	***p value***
***Sex (male)***	32.7%	44.9%	46.3%	0.259

***Age (years)***	68.5 ± 11.7	63.2 ± 14.9	63.4 ± 15.7	0.07

***Body Mass Index (kg/m*^2^)**	29.7 ± 3.8	28.05 ± 3.79		0.077

***Systolic Blood Pressure (mmHg)***	164 ± 15	132 ± 10	127 ± 13	< 0.001

***Dyastolic Blood Pressure (mmHg)***	95 ± 9	83 ± 7	79 ± 14	< 0.001

***Retinopathy ≥ 2***	80%	53%		0.02 OR 1.52 (1.12–2.07)

***Left Ventricle Hypertrophy***	75.8%	44.1%		0.001 OR 3.96 1.61–9.70

***Years of evolution***	17.05 ± 12	8.61 ± 8		0.001

The patients with resistant hypertension were treated with a mean of 3.7 drugs (100% diuretics, 50% ACE inhibitors, 68% Angiotensin II Receptor Antagonists, 70% beta blockers, 24% alfa blockers, 58% calcium channels blockers, 2% others), there were no significant differences between the resistant hypertension group treatment and the controlled hypertension group treatment.

### Distribution of the 894G>T genotypes

The allelic distribution for the healthy control group was as follows: GG 39.1%, TG 48.2% and TT 12.7%. There were no differences with the distribution of the controlled or resistant hypertension group.

In the same way, no significant association was observed when we studied the distribution of genotypes and alleles of the 894G>T NOS3 polymorphism between resistant hypertensive and controlled hypertensive groups. (Resistant hypertension GG 37.5% GT 41.7% TT 20.8% vs controlled hypertension GG 37.5% GT 48.3% TT 14.2%; p 0.47) (Table 2)

**Table 2:** 894 G>T Genotypes distribution in resistant hypertension patients and in patients with controlled hypertension.

	-894G>T NOS3				
		
	GG	GT	TT	TOTAL (n)	P
RESISTANT HYPERTENSION	18 (37.5%)	20 (41.7%)	10 (20.8%)	48	**0.47**
CONTROLLED HYPERTENSION	87 (37.5%)	112 (48.3%)	33 (14.2%)	232	

TOTAL (n)	105	132	43	280	

### Distribution of the -786T>C genotypes

The allelic distribution for the healthy control group was as follows: TT 23.6%, TC 52.7% and TT 23.6%. There were no differences with the distribution of the controlled or resistant hypertension group.

However, Analysis of the distribution of -786T>C NOS3 genotypes show that genotype -786CC is more frequent in resistant hypertension patients (Table 2). Moreover, when we distribute our population in those homozygous for the -786C allele and those carrying at least one -786T allele we observed that those homozygous for the -786C allele have higher risk of suffering resistant hypertension (Multivariate analysis: RR 2.09; 95% CI 1.03–4.24; p 0.004) (Table 3)

**Table 3:** 786T>C Genotypes distribution in resistant hypertension patients and in patients with controlled hypertension.

	-786T>C NOS3				
		
	TT	TC	CC	TOTAL (n)	P
RESISTANT HYPERTENSION	14 (29.2%)	18 (37.5%)	16 (33.3%)*	48	**0.03**
CONTROLLED HYPERTENSION	65 (28%)	126 (54.3%)	41 (17.7%)	232	

TOTAL (n)	79	144	57	280	

* ***MULTIVARIATE REGRESSION ANALYSIS***: Comparing C allele homozygotes (-786CC) and carriers of = 1 T allele (-786TT, -786TC); RR 2.09 (95% CI: 1.03–4.24) p 0.004.

## Discussion

Vascular endothelial dysfunction is a common characteristic of various cardiovascular diseases. The maintenance of regular vascular tone substantially depends on the bioavailability of endothelium-derived nitric oxide (NO) synthesized by the endothelial isoform of nitric oxide synthase (eNOS). eNOS is coded by the NOS3 gene located at 7q35. We compared the distribution of the -786T>C and G894T (Glu298Asp) NOS3 polymorphisms in patients with controlled and resistant hypertension.

The association between NOS3 gene polymorphisms and essential hypertension is not clear yet [10], and there are only a few studies investigating the association of these polymorphisms with resistant hypertension [5,11].

We did not find any difference in the distribution of genotypes of the G894T (Glu298Asp) polymorphism between the groups of patients with controlled and resistant hypertension. Thus, our results indicate that the G894T (Glu298Asp) polymorphism of the NOS3 gene did not modify the susceptibility to suffer resistant hypertension in our population. G894T (Glu298Asp) polymorphism is located in exon 7 of the NOS3 gene and has been associated with the development of hypertension [12] and endothelial dysfunction in healthy, smokers young adults [13]. Furthermore functional consequences of the Glu298Asp NOS3 variation have been demonstrated in human endothelium [14]. Nevertheless, the relation of this polymorphism with essential hypertension is not clear [15]. Recent studies described that Asp298 eNOS is more vulnerable to enzymatic cleavage in cell lysates as compared with the Glu298 protein and could potentially alter protein-protein interactions in dimer formation [16]. The only association between Glu298Asp polymorphism and resistant hypertension was reported by Jachymova et al, they found a higher frequency of the T allele in patients with hypertension and resistant hypertension compared to a control group but they did not compared hypertension versus resistant hypertension [5].

However, we found differences in the distribution of the -786T>C NOS3 genotypes being genotype CC more frequent in the resistant hypertension group. To the best of our knowledge, this is the first time that the CC genotype has been associated with resistant hypertension. Recently, this association was investigated by Sandrim et al, their results suggested that eNOS haplotypes are not associated with resistance to antihypertensive therapy [11]. However the populations investigated are different and the frequencies of the alleles even in the hypertension group are different from the ones presented in the current manuscript.

The -786CC NOS3 genotype has been associated with a decrease in the activity of the NOS3 promoter and a decrease in NOS3 mRNA levels [17]. It has been previously reported that subjects with the -786CC genotype had significantly higher systolic blood pressures and were more likely to be hypertensive [17]. Moreover, -786C NOS3 allele has been related with impaired activity of the eNOS enzyme, coronary spasm, and impaired endothelium-dependent vasodilatation in CAD patients [17,18]. On the other hand it has been reported that subjects who were homozygous for the T allele of the -786 T >C NOS3 polymorphism exhibited a significantly higher increase of forearm blood flow in response to vasodilators (acetylcholine, nitroprussiate) than CT and CC subjects [19].

Finally, in this study retinopathy and left ventricular hypertrophy were more frequent in resistant hypertension patients. These findings could be partially explained by a longer hypertension evolution in the group of patients with resistant hypertension. However it has been previously described that a variant of eNOS gene (Glu298Asp) could be an independent risk factor for left ventricular hypertrophy in human essential hypertension [20,21]. In the same way the association of eNOS polymorphisms and retinopathy has been investigated with inconsistent results [22-24].

Our data suggest that promoter variants in the NOS3 gene may play a role in the pathogenesis of resistant hypertension. We propose that the risk to suffer resistant hypertension could be determined at least partially at genetic level. However, we consider that resistant hypertension is a multifactorial syndrome and polymorphisms in other genes as well as environmental factors take part in its development.

### Limitations

This is a single-centre study with a limited number of patients; the findings of the present study should be confirmed in at least a second population. This cohort of patients has been used in other study [6]. No correction of p-values for multiple comparisons has been applied in this study.

In summary, -786CC NOS3 genotype could be a significant contributing factor to resistant hypertension and may be a genetic marker of genetic predisposition to resistant hypertension. These results further support the central role of endothelial-derived NO in the pathogenesis and response to treatment of hypertension, and could open a new perspective in the therapeutic approach of resistant hypertension.

## Conclusion

-786CC NOS3 genotype increase the susceptibility to suffer resistant hypertension and may be a genetic marker of genetic predisposition to resistant hypertension.

## Competing interests

The authors declare that they have no competing interests.

## Authors' contributions

ICG, MSL, ASR, CML and RGG have designed the study, have analyzed and interpreted the data. ICG, EC, MSL, ASR, CML and RGG have been involved in drafting the manuscript and revising it critically. ICG, MSL and EC have acquired the data. ICG, EC, MSL, ASR, CML and RGG have given final approval of the version to be published

## Pre-publication history

The pre-publication history for this paper can be accessed here:


